# Optical anisotropy of CsPbBr_3_ perovskite nanoplatelets

**DOI:** 10.1186/s40580-023-00367-5

**Published:** 2023-04-25

**Authors:** Benjamin T. Diroll, Progna Banerjee, Elena V. Shevchenko

**Affiliations:** Center for Nanoscale Materials, Argonne National Laboratory, Lemont, IL 60438 USA

**Keywords:** Perovskites, Nanoplatelets, Anisotropy, Polarization, Photoluminescence

## Abstract

The two-dimensional CsPbBr_3_ nanoplatelets have a quantum well electronic structure with a band gap tunable with sample thicknesses in discreet steps based upon the number of monolayers. The polarized optical properties of CsPbBr_3_ nanoplatelets are studied using fluorescence anisotropy and polarized transient absorption spectroscopies. Polarized spectroscopy shows that they have absorption and emission transitions which are strongly plane-polarized. In particular, photoluminescence excitation and transient absorption measurements reveal a band-edge polarization approaching 0.1, the limit of isotropic two-dimensional ensembles. The degree of anisotropy is found to depend on the thickness of the nanoplatelets: multiple measurements show a progressive decrease in optical anisotropy from 2 to 5 monolayer thick nanoplatelets. In turn, larger cuboidal CsPbBr_3_ nanocrystals, are found to have consistently positive anisotropy which may be attributed to symmetry breaking from ideal perovskite cubes. Optical measurements of anisotropy are described with respect to the theoretical framework developed to describe exciton fine structure in these materials. The observed planar absorption and emission are close to predicted values at thinner nanoplatelet sizes and follow the predicted trend in anisotropy with thickness, but with larger anisotropy than theoretical predictions. Dominant planar emission, albeit confined to the thinnest nanoplatelets, is a valuable attribute for enhanced efficiency of light-emitting devices.

## Introduction

Although all-inorganic lead halide perovskite compounds have been known for 130 years [1], interest in these compounds in both bulk and nanoscale form has expanded greatly since initial reports on the potential of related methylammonium lead iodide for photovoltaic applications [2]. Subsequently, different compositions of the CsPbX_3_ family have shown great promise in photovoltaics [3–5], downconversion [6], optical elements [7], high-energy photodetection [8], light-emitting diodes [9, 10], and lasers [11, 12]. The potential uses for CsPbX_3_ have also stimulated interest in synthetic control over the morphology of nanostructures, with the most sophisticated control achieved with CsPbBr_3_, which can be prepared in the forms of confined quantum dots of variable size [13], bulk-like cuboidal structures [6], nanowires [14], and thin plates [15–18]. Nanoplates (NPLs) of CsPbBr_3_ have a quantum well electronic structure in which the thickness dictates the band gap. These materials have been prepared with tunable quantum confinement by preparation of ensembles of 2–8 monolayers [15–19]. They are reported to have large oscillator strength [20] and narrow, polarized emission [21–23]. They have been used in light-emitting diodes [24, 25] and as optical gain media [26].

This work studies the anisotropic optical properties of CsPbBr_3_ NPLs using photoselection measurements. Previous measurements of polarization properties have focused on single particles, span limited range of energy, or do not use the same photoselection methods. Many types of semiconductor nanostructures show a strong alignment of optical polarization properties with the anisotropic shape and crystallographic axes of the material: nanorods and nanowires show strong linear polarization of photoluminescence and photodetection [27–38] whereas quantum-confined two-dimensional structures show interband optical transitions polarized in-plane [39–45]. An understanding of the transition dipole geometries coupled with control over the orientation of thin-film deposition [36, 40, 42, 46, 47] is valuable to engineering enhanced outcoupling of emitted light in LEDs, a strategy already in use for organic light-emitting diodes [48].

Here, we confirm the two-dimensional electronic structure of the CsPbBr_3_ NPLs using fluorescence anisotropy and polarized transient absorption measurements of solution samples via photoselection. These measurements show the highest optical anisotropy of the samples is observed at the lowest excitonic transition, approaching the theoretical value (0.1) of an ensemble of perfectly two-dimensional absorption and emission in the thinnest samples. All optical transitions of the NPLs at higher energies above the band edge also show in-plane absorption polarization, albeit weaker, and little sensitivity to energy. The trend toward higher fluorescence anisotropy in thinner NPLs is confirmed at the absorption band edge transition as well using polarized transient absorption measurements. In addition to the finding of two-dimensionally polarized optical transitions in CsPbBr_3_ NPLs, larger CsPbBr_3_ nanocrystals (NCs) with a cuboid shape also show a weak anisotropy. Finally, these findings are discussed in the context of predicted degeneracy-breaking of the exciton fine structure in the CsPbBr_3_ composition with non-cubic crystal structure and shape [49–52].

## Methods

### Synthesis

#### Materials and chemicals

Cesium carbonate (Cs2CO3, Aldrich, 99.9%), oleic acid (OA, Sigma-Aldrich, 90%), 1-octadecene (ODE, Sigma-Aldrich, 90%), oleylamine (Aldrich, 90%), lead bromide (PbBr2, Aldrich, 98%), mesitylene (Aldrich, 97%), ethanol (Aldrich, ≥ 99.8%), toluene (Fischer Scientific, HPLC grade), hexane (Sigma-Aldrich, ≥ 95%), dodecane (Aldrich, > 99%), and acetone (Fisher, ACS) were used without any further purification.

#### Room temperature syntheses

Room temperature synthesis of CsPbBr_3_ NPLs were performed following previous literature [53]. Precursor solutions were prepared by dissolving 32.6 mg Cs_2_CO_3_ in 10 mL oleic acid and heated under air to 100 °C until fully dissolved, then cooling to room temperature. Separately, 36.7 mg PbBr_2_ in 10 mL toluene with 0.1 mL oleic acid and 0.1 mL oleylamine was also heated under air to 100 °C until fully dissolved, then cooled to room temperature. Reactions consisted of rapid injection of cesium precursor into a solution of lead precursor, sometimes diluted with additional toluene, and then, after 10 s, the injection of acetone. Reactions proceeded for an additional 1 min, then were centrifuged for 2 min at 4000 rpm, redispersed in dodecane, centrifuged again at 2000 rpm for 3 min, and the supernatant stored for use. Samples prepared at room temperature were stored in − 80 °C freezer in dodecane to preserve the samples against degredation. Specific ratios used for different samples are shown in Table 1. It was found that the monodispersity in thickness for the samples was better for thinner samples with this method.

**Table 1:** Synthetic parameters for CsPbBr_3_ nanoplatelets synthesized at room temperature

Thickness (nm)	Cs (mL)	PbBr_2_ (mL)	Toluene (mL)	Acetone (mL)	E_gap_ (PLE, eV [nm])	E_gap_ (PL, eV [nm])
2 ML (1.2)	0.15	3.0	–	2	2.91 [426]	2.88 [431]
3 ML (2.0)	0.15	1.6	–	2	2.79 [444]	2.74 [453]
4 ML (2.4)	0.35	0.95	0.65	3	2.74 [453]	2.71 [458]
5 ML + ^a^ (3.0)	0.45	0.7	0.8	3.5	2.70 [459]	^a^2.51 [494]

^a^5ML sample contained nanoplatelets of larger thickness as well, evident in broad, redshifted photoluminescence. For PLE measurements, PL was measured at 2.67 eV (464 nm)

#### Hot injection syntheses

The synthesis for the 458 nm emitting NPLs was modified from this literature [18]. 138 mg of PbBr_2_, 1 mL of dried oleic acid, and 1 mL of dried oleylamine were loaded into a 25 mL 3-neck flask under anaerobic conditions. Mesitylene (5 mL) was added to the reaction mixture after which the flask was connected to the Schlenk line. The flask was flushed three times at room temperature by changing from vacuum to N_2_ and left on N_2_. The reaction mixture was heated to 115 °C for over an hour, and 0.8 mL of cesium oleate (prepared from 0.407 g Cs_2_CO_3_ dissolved in 21.25 mL oleic acid at 120 °C for 1 h, then held at 100 °C) was swiftly injected. 5 s following the injection, the flask was immersed in an ice-water bath to quench the reaction. Once the temperature returned to ambient, all 7.8 mL of this crude solution was centrifuged for 3 min at 5000 rpm. The resulting precipitate was dispersed in 10 mL toluene, centrifuged again for 10 min at a 11,000 rpm. The supernatant was filtered and used further for experiments. This method was found to produce purer 4 ML samples than achieved with room temperature methods, based upon the bandwidth of photoluminescence. A similar procedure was used to produce CsPbBr_3_ NCs [6], but the reaction was performed at 170 °C, using octadecene instead of mesitylene, injecting 0.4 mL of cesium oleate precursor, and cooling with a water bath.

### Steady-state spectroscopy

Absorption spectra were collected using a Cary-50 spectrometer. Photoluminescence spectra were collected using a Horiba Jobin–Yvon FL3 fluorimeter on dilute samples with 3.54 eV excitation energy. Excitation scans were made by monitoring photoluminescence intensity at a given wavelength of the emission spectrum with variable excitation wavelengths, always normalized to the intensity of the excitation source, which was monitored with a reference detector. To perform polarized photoluminescence measurements, polarizers were placed on both excitation and emission paths and four spectra were recorded corresponding to HH, HV, VH, and VV conditions. Measurement of HH and HV permits the calculation of the *g*-factor (${g=I}_{HV}/{I}_{HH}$), which describes differential throughput of the measurement optics. Polarized excitation scans were performed by collecting HH, HV, VH, and VV data for each point of an excitation scan with anisotropy (R) defined as $R\left(E\right)=\frac{{I}_{VV}-{gI}_{VH}}{{I}_{VV}+2{I}_{VH}}$. For each point, measurements were repeated until the standard deviation of the measured anisotropy was less than 7 percent.

### Time-resolved spectroscopy

Time-resolved photoluminescence was performed by exciting the samples with a weak (< 1 μJ·cm^−2^) 3.06 eV pulsed laser operated at 1 MHz, collecting the emission via fiberoptic and directing through a monochrometer to an avalanche photodiode. Polarized transient absorption spectroscopy was performed using a typical configuration for transient absorption, with a 1.55 eV Ti: sapphire amplified laser operating at 5 kHz split into a pump path, which was directed through an optical parametric amplifier to produce 3.54 eV pump photons polarized horizontal to the laser table and chopped to 2.5 kHz, and a probe path that was directed through an optical delay stage and then focused into a translating calcium fluoride crystal. The polarized pump beam and the supercontinuum white light generated from the calcium fluoride were spatially overlapped at the sample position. To ensure minimal distortion of polarization information due to the geometry of pump and probe pulses, they were configured with a small (< 5°) difference in incident angle. To discriminate between vertical and horizontal polarizations of the probe, a polarizing beam splitter was used after the sample to selectively transmit either vertical or horizontal probe light. (To ensure sufficient amounts of both, a half waveplate was used in the probe path to adjust the pump beam polarization close to the magic angle.) The transient absorption signal was generated by comparison of the pump-on versus pump-off signals of both HV and HH geometries (cross- and co-polarized, respectively). Reported measurements were collected at a fixed pump power by averaging several spectra at 10 picosecond pump-probe delay with multiple alternations of the probe polarizations.

### Structural characterization

Transmission electron microscopy (TEM) was performed on the samples using a JEOL 2100F TEM. X-ray diffraction was performed with a copper K-alpha source on a Bruker D2 phaser.

## Results and discussion

### Optical and structural characterization of CsPbBr_3_ nanoplatelets

**Fig. 1:**
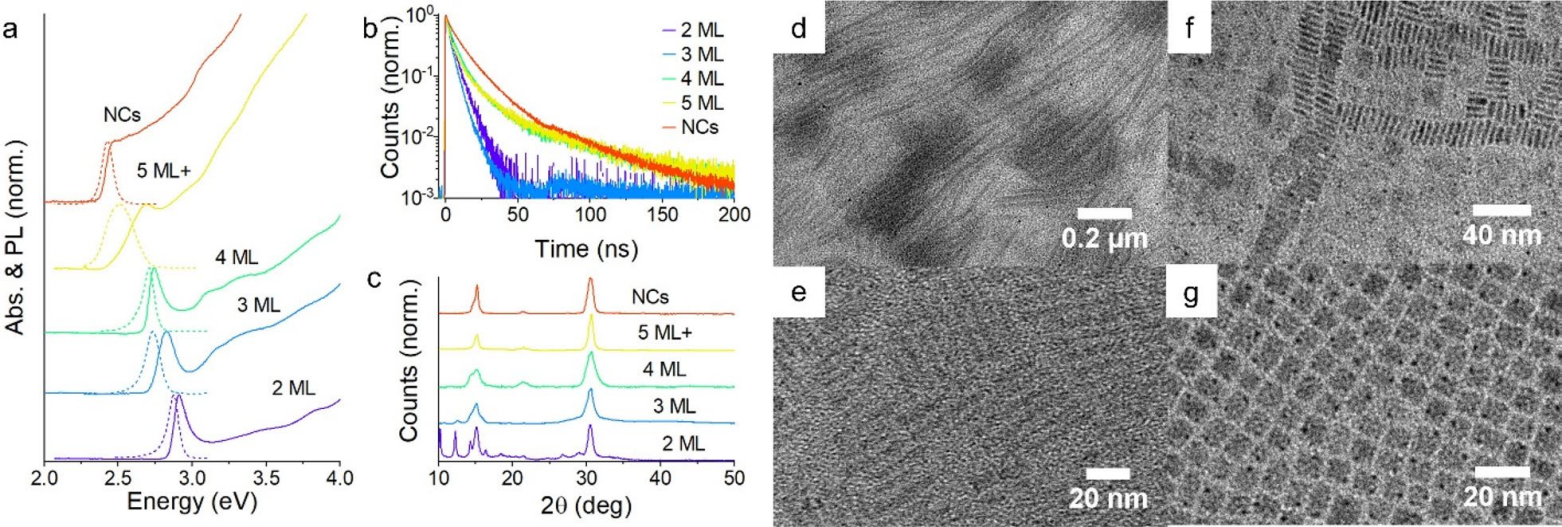
**a** Absorption (solid lines) and photoluminescence (dashed lines) of CsPbBr_3_ NPLs and NCs. **b** Time-resolved photoluminescence of the same samples. **c** Powder X-ray diffraction of drop-cast solids of the same samples. **d** Lamellar strands of 2 ML NPLs at low magnification. **e**, **f** Higher magnification images of **e** 3 ML NPLs, **f** 4 ML NPLs, and **g** NCs

The CsPbBr_3_ NPLs and NCs used in this work are characterized with data in Fig. 1. Samples of NPLs were isolated with absorption spectra (shown in solid lines) which were previously identified in literature as 2 monolayer (ML) to 5 ML in thickness [19, 53]. 1 ML samples, which can be prepared synthetically, evidenced by a sharp absorption feature near 400 nm, were unstable at room temperature for the time necessary to perform other data collection. As stability is an acute concern with CsPbBr_3_ NPLs, samples measured to ensure matching absorption before and after measurements. The absorption spectrum of a larger, cuboid CsPbBr_3_ NC sample is also shown and used for comparison throughout this work. With increasing thickness, the sample absorption red-shifts to smaller band gap and converges in the case of the NC sample with the bulk value for CsPbBr_3_. Photoluminescence (dashed lines in Fig. 1a) data also show this same shift, although they also indicate the presence of impurities in the thickest 5 ML NPL ensemble; these may come from thicker NPLs or other NCs with a smaller band gap (These impurities do not strongly influence the following analysis, because emission from 5 ML samples can be isolated spectrally). The photoluminescence lifetime of the samples shown in Fig. 1b increased to longer lifetimes for the thicker NPLs and NC samples, but in all cases the lifetime remains less than 20 ns, which is important for anisotropy measurements as photon emission is assumed to occur on a faster time-scale than orientational changes of the emitter. Given the size of the NPLs or NCs used in this work, rotational diffusion is substantially slower (hundreds of nanoseconds to microsecond) [54] than light emission.

The X-ray diffraction patterns for the samples in Fig. 1c show the same primary diffraction features across all the samples, at 15° and 31° in 2θ. The exact crystal structure of nanosized CsPbBr_3_ was a subject of several investigations: the lowest symmetry orthorhombic crystal structure is now typically understood to be the crystal structure observed in CsPbBr_3_ NCs under standard conditions [55–57], although there are still differences reported at the level of individual NCs analyzed by electron microscopy [58]. The orthorhombic crystal structure is apparent from both small peaks from the reduced symmetry of the crystal as well as shoulders or asymmetric reflection features. The same distortions are observed in NPLs of CsPbBr_3_ and previous works have shown that such NPLs are also orthorhombic in crystal structure, indicating a small symmetry breaking of the in-plane axes [18, 59]. Importantly, the emergent diffraction peaks associated with zero-dimensional Cs_4_PbBr_6_, previously reported from NPL instability, are not observed in the sample [60]. The changes in the peak ratios across the samples most likely results from anisotropic orientation of the drop-cast samples. The only substantial deviation in the X-ray diffraction signals in Fig. 1c is the additional peaks at low angles for the 2 ML sample: this sample has a series of reflections at 10.2, 12.3, 14.4, 16.4, and 18.4 degrees in two-theta, which occur from the lamellar spacing (~ 3.9 nm) of stacks of NPLs in the solid state. This provides additional confirmation that the NPL geometry is preserved and the NPLs have not transformed in shape. A microscopic transmission electron microscopy (TEM) images of such stacks is shown in Fig. 1d–f and in previous literature [17, 59]. Although stacking of NPLs is well-known to occur in other systems (e.g. CdSe NPLs [61, 62]), its prevalence in solutions of CsPbBr_3_ NPLs is not clear from the solid film X-ray diffraction and microscopy. The TEM images of the samples of NPLs otherwise confirm their anisotropic shape and the cuboid structure of the CsPbBr_3_ NCs.

### Fluorescence anisotropy

To measure the anisotropic optical properties of the CsPbBr_3_ NPLs and NCs, fluorescence ansiotropy experiments were employed on ensembles of particles in solution [63]. This technique has an extensive track record in evaluation of polarized optical properties of nanomaterials of many different geometries including nanorods and NPLs [28, 30–32, 39, 40]. These measurements employ a polarized excitation or pump source (vertical or horizontal) to photoselect a sub-population of the ensemble and then measure the emitted polarization (at 90°) of the sample through a second polarizer. By collecting four spectra (HH, HV, VH, and VV) a value of anisotropy can be obtained from the measurements. Anisotropy (R) is.

$$R=\frac{{I}_{VV}-{gI}_{\mathrm{VH}}}{{I}_{VV}+{2gI}_{\mathrm{VH}}}=\frac{{I}_{\| }-{I}_{\perp }}{{I}_{\| }+{2I}_{\perp }},$$

in which $g={I}_{HV}/{I}_{HH}$ is a factor adjusting for the differential throughput of vertical and horizontal polarizations through collection optics. Anisotropy of random ensembles has a full range of − 0.2 to 0.4. The virtue of anisotropy, compared to polarization—another common metric—is that *R* for an ensemble is the weighted average of anisotropy values of its constituents.

**Fig. 2:**
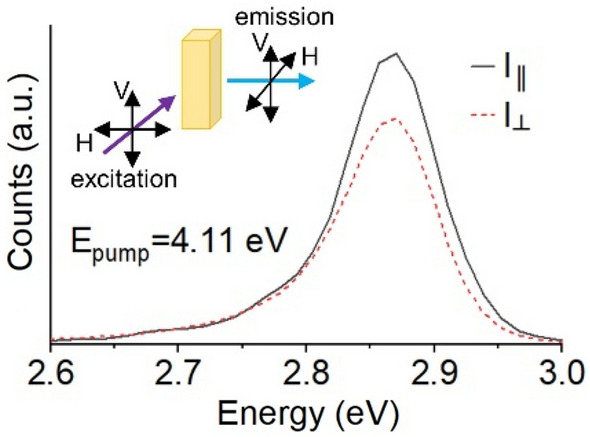
Photoluminescence of 2 ML CsPbBr_3_ NPLs for different pump energies and polarizations. Inset is a cartoon of the measurement conditions: measurements are performed on toluene solution ensembles, using polarized vertical or horizontal excitation and vertical or horizontal polarization of the emitted photoluminescence at 90° from the pump excitation

An example of the measurement configuration and measurement data polarization-dependent photoluminescence spectra is shown in Fig. 2. Here, an ensemble of 2 ML CsPbBr_3_ NPLs is excited at a single energy of 4.11 eV (300 nm). The intensity of the co-polarized emission (${I}_{\| }$) is more intense than the cross-polarized emission (${I}_{\perp }$) indicating a positive anisotropy for the sample. The ratio of the integrated counts of the two photoluminescence spectra yields an anisotropy of 0.067. This number is similar to values for the measured anisotropy of CdSe NPLs with excitation far above the band gap [40, 64], but lower than typical values of nanorods [28, 30–32]. The hypothesized reason for the lower anisotropy values and their fundamental limits in two-dimensional samples will be discussed in Sect. 3.4.

**Fig. 3:**
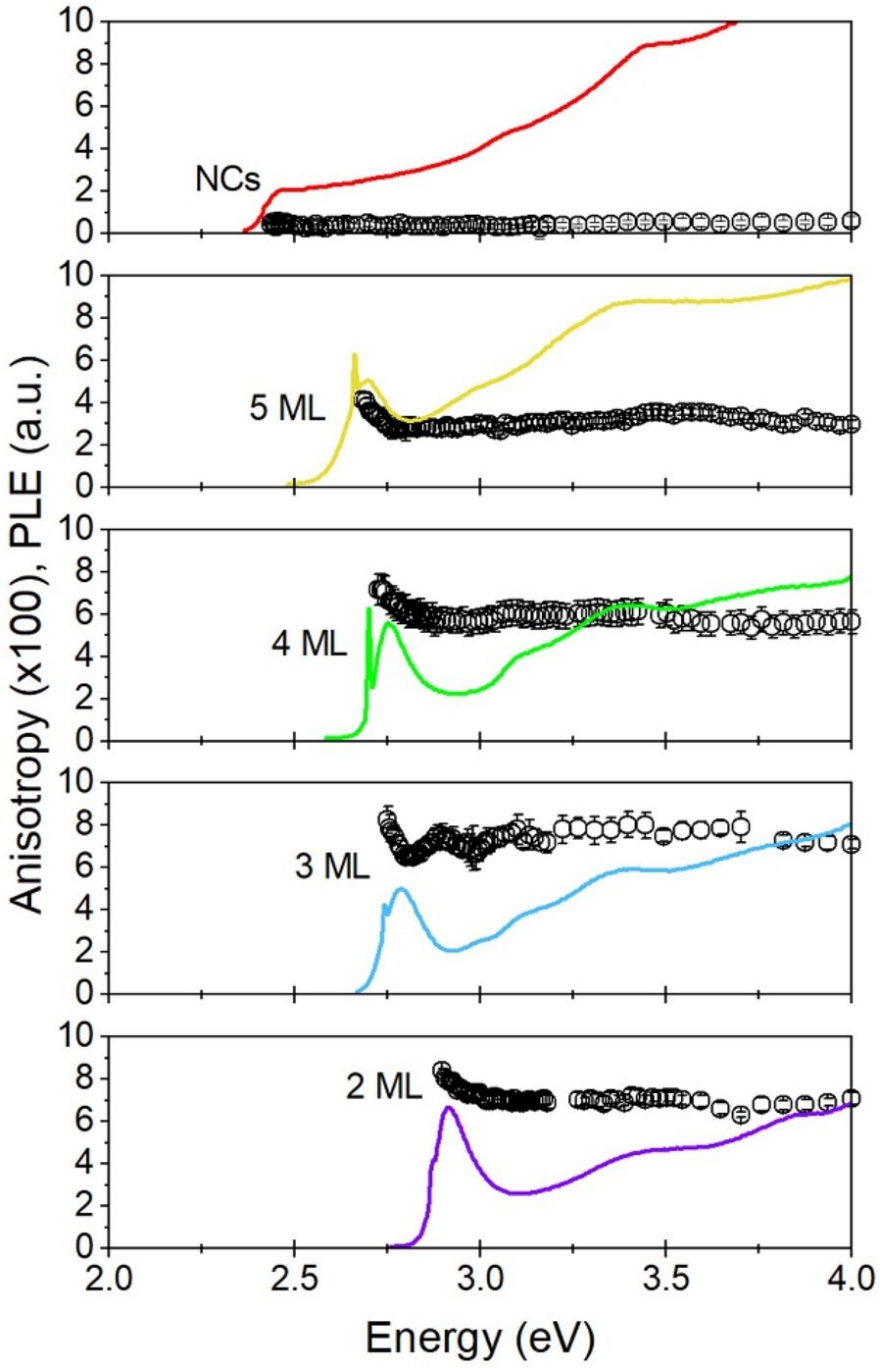
Photoluminescence excitation scans (solid lines, arbitrary scale) and energy-dependent fluorescence anisotropy (open circles). Each data point represents the calculated fluorescence anisotropy of at least 3 measurements measured with an excitation energy of the data point. Anisotropy values are shown scaled (× 100), but the same scale is used for each panel

The measurement technique in Fig. 2 is extended to the photoluminescence of all samples with variable excitation energies in Fig. 3. Photoluminescence excitation permits the sampling of only part of the photoluminescence curve, which enables selection of a sub-set of the ensemble and overcomes the poor monodispersity evident in the absorption spectrum of the 5 ML sample by selectively examining only the NPL constituents. The solid, colored lines indicate the (unpolarized) photoluminescence excitation data for the samples show strong excitonic absorptions indicative of a monodisperse population of CsPbBr_3_ NPLs which is sampled in the excitation measurement. In most cases, a small spike from scattered light is observed at the excitation energy which is monitored. Data for anisotropy of each of the samples of CsPbBr_3_ NPLs and NCs are shown in black open circles, with error bars indicating the standard deviation of the measured value. Data from the points with scattered light are not included in the reported anisotropy because scattered light (for which R = 1) contributes artificially to measured anisotropy. The results in Fig. 3 converge with the observed anisotropy of the same sample reported in the polarized emission spectra of Fig. 2: measuring the anisotropy of for many high-energy excitation wavelengths shows an asymptotic value at high energy of c. 0.065. Each of the NPLs samples also shows a similar flattening of the anisotropy spectrum at high excitation energies, a feature which is common in measurements of nanorods and other NPLs as well. At lower energies, near the band edge absorption feature, the anisotropy observed for each of the samples increases to the highest value observed of all excitation energies, a feature which is also typical of earlier measurements of anisotropy of nanorods and NPLs. This pattern of higher anisotropy at the band edge and a tapering to asymptotic values at high energy is understood to originate from electronic and dielectric contributions to anisotropy, respectively [28, 30]. At low energies near the band edge, the absorption transitions of the lowest excitonic states of the NPLs are well-separated from other absorption transitions and therefore the signal near the band edge reflects absorption and emission from only from band edge excitonic state. It is therefore the anisotropy near the band edge which provides the most information on the polarization properties of the unique quantum confined states of the NPLs. Although emission is always derived from the same transitions, at higher excitation energy, continuous two-dimensional electronic states and higher excitonic absorptions contribute to the absorption and therefore absorption anisotropy. The absorption anisotropy at energies far above the band gap converges with the expected anisotropy from dielectric screening of light, which is weaker in the shorter axis of a sample [65].

Among the measured samples, there is a trend toward lower values of anisotropy both at the band edge and at high-energy as the samples of NPLs become thicker. This continues for the NC sample. However, the NC sample shows anisotropy values that are nearly flat, but positive. Anisotropy from a symmetric sample of CsPbBr_3_ is not expected from either electronic or dielectric contributions but may be observed due to the broken symmetry of the crystal structure of slight anisotropy of the cuboid shape (see discussion below). Indeed, TEM imaging of the projections of the NC sample shows a small anisotropy of the dimensions with a length ratio of 1.2. It is noteworthy that earlier measurements of CsPbBr_3_ cuboids have also shown weakly polarized optical transitions [66–68].

### Polarized transient absorption

**Fig. 4:**
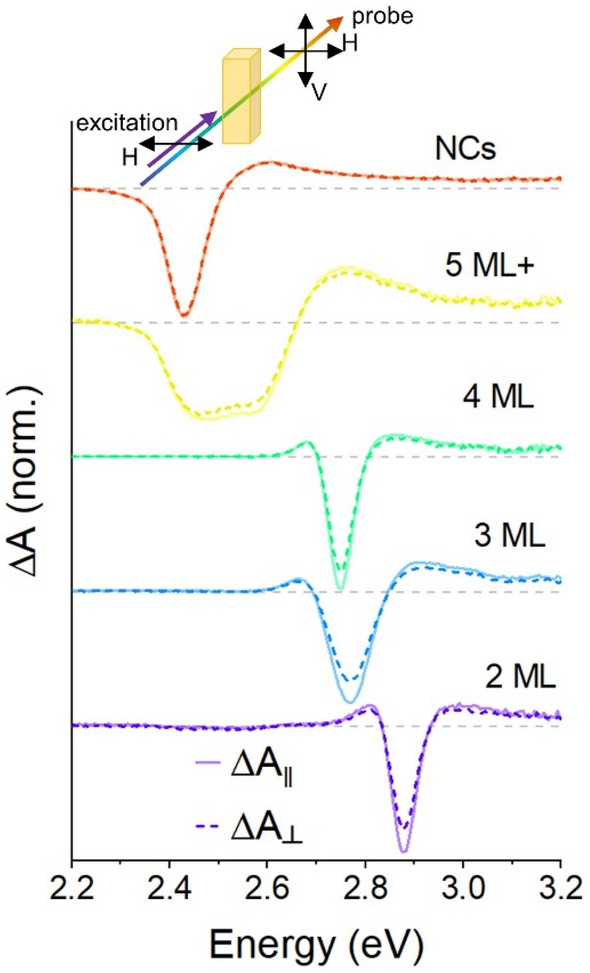
Polarized transient absorption spectroscopy for NPLs and NCs with data showing the transient absorption spectrum at 10 ps pump-probe delay with 3.54 eV pump photon energy. The inset shows the geometry of the measurement; a horizontally-polarized pump excites the toluene-dispersed samples and vertical and horizontal probe components are separated after the sample using a polarizer. ΔA signals are shown co-polarized (solid lines) and cross-polarized (dashed lines)

A second measurement technique which has been used to measure anisotropy of colloidal nanoparticles is polarized transient absorption spectroscopy [34, 37]. In these measurements, a polarized pump excitation also photoselects the randomly-oriented ensemble in solution and a second probe pulse measures change in the light transmission through the sample under co- or cross-polarized conditions (see inset cartoon on Fig. 4). Anisotropy may be calculated by comparison of the transient absorption spectra (ΔA) with different probe polarizations. Figure 4 shows such transient absorption spectra of the samples for both co-polarized (solid lines) and cross-polarized (dashed lines) pump and probe pulses, using a 3.54 eV pump energy. To ensure that data were collected after electron–phonon coupling was complete and there are no non-equilibrium contributions from electrons, [69, 70] a pump-probe delay of 10 ps was used for these spectra. As anticipated, the spectral responses are nearly identical: a strong negative ΔA known as a bleach occurs at the band edge absorption of the samples, indicating filling of the band edge states and weakening of absorption. A small positive ΔA signal (induced absorption) is observed just below the band edge for the NPL samples, which is attributable to biexciton absorption [70].

As anticipated based upon the fluorescence anisotropy measurements shown in Sect. 3.2, the intensity of ${\Delta \mathrm{A}}_{\| }$ signals is greater than the intensity of ${\Delta \mathrm{A}}_{\perp }$, particularly for the 2–4 ML NPL samples. Like the photoluminescence data in Fig. 2, the anisotropy of the polarized transient absorption signals can be calculated from their intensity.

$$R=\frac{{\Delta A}_{\| }-{\Delta A}_{\perp }}{{\Delta A}_{\| }+2{\Delta A}_{\perp }}$$

**Fig. 5:**
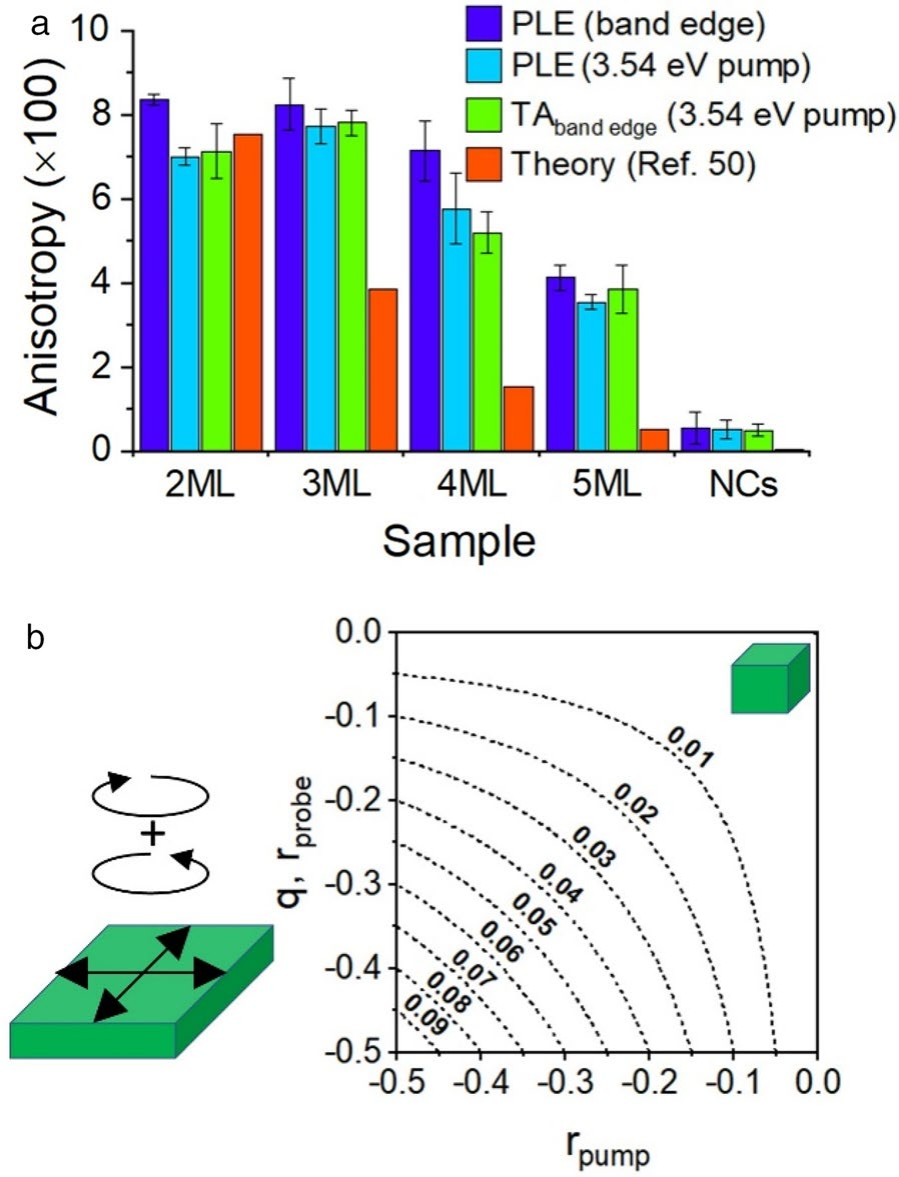
**a** Summary of optical ansiotropy results for the CsPbBr_3_ NPLs and NCs. Bar graphs of photoluminescence excitation (PLE) data and transient absorption (TA) data are shown. **b** Plot of the theoretical values of photoselection anisotropy of an ensemble of two-dimensional emitters with larger, degenerate dipole strength in-plane, or, equivalently, circular-polarized optical transitions

Here, the peak absolute values of $\Delta \mathrm{A}$ are used to calculate anisotropy because the zero-crossing points of transient absorption data generate indeterminate values. This also reduces the influence of noise. Calculating the anisotropy accordingly yields a value of 0.071 for the 2 ML sample. (Results from other samples are catalogued in Fig. 5).

Similar to the photoluminescence excitation data, the anisotropy observed for the NPL samples decreases successively for the 4 and 5 ML samples compared to the 2 and 3 ML samples, with the NCs showing the lowest anisotropy overall. Polarized transient absorption measurements confirm that the band edge absorption and band edge emission of the NPLs have similar degrees of anisotropy. An additional value added by transient absorption spectroscopy compared to the fluorescence spectrum is that it probes absorption transitions with both pump and probe pulses and therefore elucidates more information about absorption anisotropy away from the band edge. In particular, it is noted that although the bleaching of the optical transition at the band edge yields values of anisotropy which are comparable to those from high-energy polarized photoluminescence excitation data. However, the ΔA signals at higher energy than the band edge bleach (e.g. > 3.1 eV) show weaker sensitivity to polarization. Such higher-energy absorption transitions are associated with higher quantum well levels or non-excitonic absorption of the two-dimensional well.

### Two-dimensional polarization and exciton fine structure

The data from both band edge and high energy fluorescence anisotropy measurements and polarized transient absorption measurements is summarized in Fig. 5. The thinner (2 ML and 3 ML) NPLs show values of anisotropy approaching 0.1, particularly at the band edge where polarization properties are normally strongest. The thicker (4 ML, 5 ML) NPLs show a progressive decrease in the measured anisotropy and cuboidal CsPbBr_3_ NCs show small but consistently positive values of anisotropy by all measurements. To interpret these results, it is helpful to provide a framework of the origin of photoselection anisotropy in materials systems with a unique axis ($x=y\ne z$), such as NPLs or nanorods. Here, the absorption or emission transitions can be described by how transition intensity maps on to the sample axes (e.g. $\left({r}_{x},{r}_{y},{r}_{z}\right)$ for absorption) normalized such that ${r}_{x}+{r}_{y}+{r}_{z}=1$ and constrained with ${r}_{x}={r}_{y}$, which permits definition of absorption defined as $r=\frac{{r}_{z}-{r}_{x}}{{r}_{z}+2{r}_{x}}$, with analogous expression for emission transitions, and a range of -0.5, for perfect in-plane polarization, to 1.0, for perfect out-of-plane polarization. Following convention, the absorption transition anisotropies are labeled for pump excitation (r_pump_) or probe (r_probe_), and the emission anisotropy as q. With these stipulations, anisotropy (R) of a randomly oriented ensemble is a convolution of the anisotropy of the optical absorption transition (r_pump_) and the anisotropy of the emission (q) or absorption (r_probe_) the optical transition in fluorescence or transient absorption, respectively:

$$R\left(E\right)=\frac{2}{5}{r}_{pump}\bullet q \mathrm{or }R\left(E\right)=\frac{2}{5}{r}_{pump}\bullet {r}_{probe}$$

A contour map of the potential values of anisotropy for optical transitions polarized in-plane (e.g. $\left({r}_{x}={r}_{y}>{r}_{z}\right)$) is show in Fig. 5b. It can be seen from the above equations that the similarity of fluorescence anisotropy collected with 3.54 eV pump, which convolves absorption and emission transitions, and polarized transient absorption at 3.54 eV pump, which convolves two absorption transitions, confirms that the band edge absorption and emission have similar symmetry.

Further, the contour plot in Fig. 5b provides instructive guidance on the range of absorption or emission polarization which are possible for the NPL samples. For example, the band-edge fluorescence anisotropy of the 2 ML sample, which reaches 0.086, means that both r_pump_ and *q* $\le -0.43$, with *q* ordinarily assumed to be smaller than r_pump_ [28]. Thus, emission polarization, another common metric defined as $P=\frac{{I}_{\| }-{I}_{\perp }}{{I}_{\| }+{I}_{\perp }}$, is *at least* 0.82 for the 2 ML sample, with progressively lower emission polarization for thicker samples. The strength of planar absorption and emission transitions of the CsPbBr_3_ NPLs is similar to other colloidal quantum wells [40, 41, 64, 71]. The emission polarization estimated here for the thinnest NPLs, $\ge 0.75$, exceeds the reports for ensembles of CsPbBr_3_ NPLs, which suggest imperfect alignment in earlier measurements [21, 72]. However, individual CsPbBr_3_ NPLs have been studied by polarized single particle spectroscopy with values at low temperature of emission polarization of c. 0.8, which is quite close to the predicted polarization here [22]. These results are also similar to those from bulk single crystals of two-dimensional materials with similar compositions, in which in-plane emission dipole strength is reportedly up to 90% in plane (i.e. $P=0.8$ or $q=-0.4$) [73]. Thus these results underline that with control over assembly orientation, which has been achieved in nanorod [40, 74–77] and other NPL systems, [42, 46, 47] macroscopic polarization critical for enhanced efficiencies in light-emitting devices can be obtained from CsPbBr_3_ NPLs. This strong planar polarization is sequentially diminished for the thicker NPLs examined and the NCs, which indicates that the wavefunctions of the NPLs evolve substantially toward isotropic transitions with thickness.

To explain these observations, it is useful to consider that plane-polarized emission and absorption can have electronic origins, due to unique symmetry of quantum confined electronic structure, or dielectric origin, arising from shape. As described above, electronic effects on the NPL anisotropy are greatest at the band edge, depending on exciton fine structure. Previous works have described the symmetry of the dominant transitions of the band edge excitons through calculations of the exciton fine structure [49, 50, 78, 79]. These calculations have underlined how the reduced symmetry of the orthorhombic crystals of NCs and the planar extension of NPLs breaks the symmetry of electronic transitions [51, 52, 78]. Crucially, the crystal structure and shape of the NPLs yields a splitting of the energies between optically-allowed excitonic transitions which are polarized in-plane versus those polarized along the unique axis. Plane-polarized transitions may be characterized by the near degenerate orthogonal transitions shown in Fig. 5b, but a different understanding consistent with exciton fine structure calculations is that the planar absorption and emission transitions of the band edge excitons are circularly-polarized [49, 50]. Calculations suggests that excitonic states which are polarized in-plane are 5–16 meV lower in energy than bright emissive states polarized along the NPL unique axis [50]. Larger energetic spacings are calculated for thinner NPLs, which relates directly to the observed dependence with thickness: photoluminescence of the NPLs at room temperature will reflect the oscillator strength and thermal occupation of allowed optical transitions, with weaker polarization of thicker samples. The anisotropy factor of the optical transitions is also predicted to diminish substantially between 2 and 5 monolayers, as shown in the orange bars in Fig. 5a [50]. The experimental results of this work be understood as a verification of the calculations of exciton fine structure showing strong in-plane polarization of the thinnest NPLs, with diminishing values at thicker size. However, the experimentally observed trend in diminishing anisotropy is not as rapid with NPL thickness as predicted theoretically. This is most clear in the similarity of the 2 ML and 3 ML samples; despite predictions of substantially lower optical polarization, the 3 ML sample even shows slightly higher anisotropy at 3.54 eV pump. This may be attributed to the excitonic absorption structure of the respective samples: the samples show small increases in anisotropy associated with the excitonic absorptions in the PLE spectrum. Finally, the finding of anisotropy of the more symmetrical cuboid NCs is also consistent with relatively weak polarization of the exciton fine structure from the orthorhombic crystal and anisotropy of the cuboid structure itself [51, 52, 78].

## Conclusions

In conclusion, the data presented in this work have shown that the optical properties of CsPbBr_3_ NPLs are highly-polarized in the long plane of the NPLs. Fluorescence ansiotropy measurements indicates that the band-edge absorption and emission for ensembles of NPLs exceeds 0.08, approaching the theoretical value of perfect in-plane polarization of 0.1. The degree of optical anisotropy is, however, strongly-dependent on the thickness of the NPLs, with diminishing anisotropy observed for thicker NPL samples and still weaker ansiotropy in cuboidal, larger CsPbBr_3_ NCs. The results confirm the predictions of exciton fine structure calculations of a strong thickness-dependence of anisotropy, which stands in contrast to other NPL systems. These results convey that thin CsPbBr_3_ NCs, particularly 3 ML and fewer, are excellent candidates for polarized blue light emitters and incorporation into aligned layers in light emitting technologies.

## Data Availability

The datasets used and/or analysed during the current study are available from the corresponding author on reasonable request.
