# Correction: Optical anisotropy of CsPbBr_3_ perovskite nanoplatelets

**DOI:** 10.1186/s40580-023-00391-5

**Published:** 2023-09-11

**Authors:** Benjamin T. Diroll, Progna Banerjee, Elena V. Shevchenko

**Affiliations:** Center for Nanoscale Materials, Argonne National Laboratory, Lemont, IL 60438 USA

**Correction: Nano Convergence (2023) 10:18** **https://doi.org/10.1186/s40580-023-00367-5**

In the original publication of the article [1], the copyright holder was incorrectly given as ‘© Argonne National Laboratory, under exclusive licence to Korea Nanotechnology Research Society (KoNTRS) and Springer Nature Singapore Pte Ltd. 2023' but should have been ‘© Argonne National Laboratory'. This has now been corrected with this erratum.

The original article has been corrected.
